# Immobility, inheritance and plasticity of shape of the yeast nucleus

**DOI:** 10.1186/1471-2121-8-47

**Published:** 2007-11-09

**Authors:** Thomas Hattier, Erik D Andrulis, Alan M Tartakoff

**Affiliations:** 1 Cell Biology Program, Case Western Reserve University, 10700 Euclid Avenue, Cleveland, OH, 44106 USA; 2 Department of Pathology Case Western Reserve University, 10700 Euclid Avenue, Cleveland, OH, 44106, USA; 3 Department of Molecular Biology and Microbiology, Case Western Reserve University, 10700 Euclid Avenue, Cleveland, OH, 44106, USA

## Abstract

**Background:**

Since *S. cerevisiae *undergoes closed mitosis, the nuclear envelope of the daughter nucleus is continuous with that of the maternal nucleus at anaphase. Nevertheless, several constitutents of the maternal nucleus are not present in the daughter nucleus. The present study aims to identify proteins which impact the shape of the yeast nucleus and to learn whether modifications of shape are passed on to the next mitotic generation. The Esc1p protein of *S. cerevisiae *localizes to the periphery of the nucleoplasm, can anchor chromatin, and has been implicated in targeted silencing both at telomeres and at HMR.

**Results:**

Upon increased Esc1p expression, cell division continues and dramatic elaborations of the nuclear envelope extend into the cytoplasm. These "escapades" include nuclear pores and associate with the nucleolus, but exclude chromatin. Escapades are not inherited by daughter nuclei. This exclusion reflects their relative immobility, which we document in studies of prezygotes. Moreover, excess Esc1p affects the levels of multiple transcripts, not all of which originate at telomere-proximal loci. Unlike Esc1p and the colocalizing protein, Mlp1p, overexpression of selected proteins of the inner nuclear membrane is toxic.

**Conclusion:**

Esc1p is the first non-membrane protein of the nuclear periphery which – like proteins of the nuclear lamina of higher eukaryotes – can modify the shape of the yeast nucleus. The elaborations of the nuclear envelope ("escapades") which appear upon induction of excess Esc1p are not inherited during mitotic growth. The lack of inheritance of such components could help sustain cell growth when parental nuclei have acquired potentially deleterious characteristics.

## Background

The position, shape, size and orientation of organelles varies among differentiated cells, thereby allowing distinct cell types to be recognized. The approximately spherical nucleus itself generally is near the center of the cell and in yeasts the spindle pole body (SPB) provides a landmark at one pole of the nucleus, thereby allowing one to witness changes of nuclear orientation during the cell cycle [1,2]. Related to these observations is the question of whether all portions of the nuclear envelope (NE) are necessarily inherited by the daughter nucleus at mitosis – an issue which bears directly on the ability of cells to cope with the impact of damage or change accrued during a single generation. The present investigation shows that a novel landmark of the perimeter of the yeast nucleus is not inherited.

The nuclear lamina in higher eukaryotic cells governs nuclear morphology and serves as a scaffold for the organization of the nucleoplasm, where it anchors heterochromatin and can affect both DNA replication and transcription. The best-characterized proteins of the lamina are the intermediate filament lamins, which self-associate *via *coiled-coil domains, generating a compact meshwork at the nuclear periphery [3].

Alterations in the structure, organization, and composition of the nuclear lamina are likely to account for aberrant shapes of the nuclei of malignant cells, as well as the distinct nuclear morphology of neutrophils. Moreover, the increased titer of mutant lamins can have major consequences for the shape and integrity of the nuclear surface [3-6]. Lamin orthologs are absent from yeast, and it is not known whether a structural or functional equivalent of the lamina exists in *S. cerevisiae*. When higher eukaryotic lamin B1 or its receptor is expressed in *S. cerevisiae*, these proteins concentrate at the periphery of the nucleoplasm [7].

Several non-membrane proteins that normally localize to the periphery of the nucleoplasm in yeast possess coiled-coil domains (Esc1p, Mlp1/2p, Sir4p, Smc5/6p) [8-11]. Esc1p (Establishes Silent Chromatin), contains three equally spaced coiled-coil domains, can function as an anchor for chromatin, interacts with Rap1p and Sir4p, promotes targeted silencing at telomeres and HMR, is needed for proper organization of the "nuclear baskets" of nuclear pores, and promotes nuclear retention of unspliced transcripts [8,12,13]. Esc1p is larger than lamins (187 *vs *70 kDa) and lacks the C-terminal isoprenylated CAAX motif present in A and B-type lamins.

The present study demonstrates that accumulation of Esc1p at the nuclear periphery causes dramatic modifications of the nuclear envelope. These modifications are structurally distinct from those which have been reported in a screen of deletion strains [14] or upon deletion of an ER/NE membrane phosphatase [15-17], mutation of components of the nuclear pore complex [18], proteins which function in the early secretory path [19,20], or Acc1p, which reduces very long-chain fatty acids [21]. Interestingly, the titer of Esc1p affects levels of several transcripts, only some of which originate from telomere-proximal loci. The structural abnormalities of the NE which are caused by Esc1p are not passed on to daughter cells, showing that inheritance of components of the nuclear perimeter can be selective.

## Results

**Figure 1:**
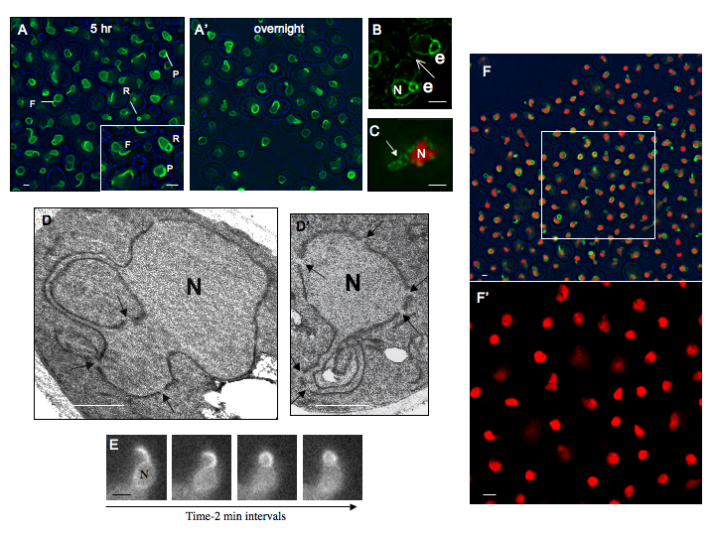
**Structure of Escapades**. **(A) (A') **Haploid cells expressing GFP-Esc1p from a galactose-inducible promoter (ATY3258) were induced for 5 hrs (left) or overnight (right) with 2% galactose. Escapades are seen as "fins" (F), "rings" (R) or "patches" (P). Phase images are in blue. Induction with 10–100-fold lower concentrations of galactose reduced the number of cells which were fluorescent but did not affect the appearance of their escapades. Note that fewer fins are seen upon overnight induction. The scale bar in this and all figures designates 1 micron. **(B)** Strain ATY2957 expressing both untagged Esc1p from a galactose-inducible promoter and a GFP-tagged ER marker (a truncated form of HMG-CoA reductase 1-GFP [59]), after overnight induction. Two cells are illustrated – one toward the upper right and one in the lower middle portion of the field. Note the ring-like escapades (e) in both cells. Fin-like escapades are seen after shorter periods of induction and no such structures are seen in the absence of galactose. The peripheral ER is indicated by the arrow. N: The nucleoplasmic volume. **(C)** Cells which express mRFP-tagged Htb2p and Nup49p-GFP as well as galactose-inducible untagged Esc1p (ATY3156) were induced overnight. Note the presence of the Nup49p-GFP signal (arrow) outside the margin of the red chromatin mass. By contrast, controls in glucose medium show a conventional circumferential distribution of GFP signal (not shown). **(D) (D') **GFP-Esc1p-expressing cells (ATY1483) were induced overnight, fixed and processed for transmission EM. Note the constant width of the double membranes that extend from the surface of the nucleus, and the presence of nuclear pores in these extensions (arrows). **(E) **Time-lapse sequence of a GFP-Esc1 expressing strain (ATY2102) after overnight induction. Note the progressive conversion of the fin to a ring-like escapade. Such conversions are seen only infrequently. N: The spherical portion of the nucleus. **(F) (F') **Htb2p-mRFP-expressing cells induced for 5 hr to express GFP-Esc1p (ATY3281). The square in the upper field is enlarged and illustrated without the green signal below to clarify the distribution of Htb2p-mRFP. Note the absence of tagged chromatin from the escapades.

To learn whether the composition of the nuclear periphery influences nuclear shape, and to extend previous observations on Esc1p [8], we have induced synthesis of GFP-tagged Esc1p in *S. cerevisiae*. After 3–5 hrs we observe the progressive elaboration of fin- and ring-like "escapades" that extend from the surface of > 80% of nuclei. ~6% of nuclei also have bright patches of GFP-Esc1p at the nuclear periphery. After overnight induction, rings and patches predominate (Fig. 1A). Similar observations have been made with both haploid and diploid strains. Equivalent observations have also been made with untagged Esc1p, in which case we visualize a GFP-tagged ER membrane protein to define the perimeter of the nucleus (Fig. 1B). Judging from estimates of transcript levels presented below, the titer of Esc1p could increase as much as 20–30x over controls upon overnight induction.

Images of cells which express Nup49p-GFP (Fig. 1C) show that escapades include nuclear pores (Fig. 1C). Ultrastructural examination also detects nuclear pores in the escapades and demonstrates that escapades are double-membrane sheets (rather than tubules) of constant width (Fig. 1D). Since escapades are limited by two layers of NE it is reasonable that – in cells expressing GFP-Esc or ER membrane proteins – their fluorescent intensity can exceed that of the rest of the NE (Fig. 1A/B). Time-lapse observations show that the position and contour of most escapades remains essentially constant for tens of minutes; however the fin-like structures occasionally fuse back to the nucleus and generate rings (Fig. 1E). Examination of cells which express a tagged histone (Htb2p-mRFP) as well as GFP-Ec1p shows that escapades include little or no chromatin (Fig 1F).

To learn whether excess of other proteins of the nuclear periphery causes similar changes, we have compared the impact of excess Esc1p to that of the non-membrane protein, Mlp1p [See Additional file 1] which colocalizes with Esc1p [9,22], the tail-anchored inner membrane protein, Prm3p [23] [See Additional file 2], and the integral membrane proteins of the inner membrane, Heh1p and Heh2p [24] [See Additional files 3 and 4]. Excess Mlp1p is known to distribute throughout the nucleoplasm [9]. As shown, Mlp1p has no obvious effect and Prm3p causes changes of the NE roughly comparable to those caused by Esc1p. By contrast, the Heh proteins are much more perturbing, with Heh1p not even leaving the quasi-spherical shape of the chromatin mass intact. Induction of either Esc1p or Mlp1p allows continued growth, while induction of Heh1p, Heh2p, or Prm3p is toxic (not shown). We therefore have not pursued these membrane proteins further.

### Relation of Escapades to Intranuclear Structures

**Figure 2:**
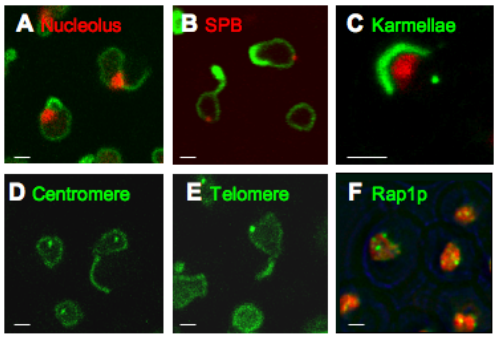
**Relation to Nuclear Structures**. **(A) **GFP-Esc1p was induced for 5 hr to compare the localization of GFP-tagged escapades to the nucleolus, in a strain (ATY2101) which expresses Sik1p-mRFP. Systematic examination of through-focal series shows that > 90% of escapades contact the perimeter of the nucleus at or immediately adjacent to the mRFP-positive nucleolus. See Fig. S5. **(B) **Comparison of the localization of GFP-tagged escapades with the spindle pole body, in a strain (ATY3276) which expresses Spc42p-mRFP and has been induced for 5 hr. An overview is given in Fig S6. **(C) **Comparison of the localization of GFP-tagged karmellae with the nucleolus and the spindle pole body, in a strain (ATY1577) which expresses Sik1p-mRFP and Spc42p-GFP and carries plasmid [pGAL-HMG-CoA Reductase I-GFP]. Karmellae are ER membrane stacks associated with the outer nuclear membrane which result from overexpression of HMG-CoA reductase type1. **(D) **Comparison of the localization of GFP-tagged escapades with a centromere, in a strain (ATY2098) which expresses a GFP-lac repressor fusion and an insertion of lac operator arrays near CENIV and has been induced for 5 hr. An overeview is given in Fig. S7. **(E) **Comparison of the localization of GFP-tagged escapades with a telomere, in a strain (ATY2097) which expresses a GFP-lac repressor fusion and carries an insertion of lac operator arrays near telomere XIVL and has been induced for 5 hr. An overview is given in Fig. S8. **(F) **Comparison of the localization of the GFP-tagged telomere-associated protein, Rap1p, and Htb2p-mRFP-tagged chromatin in ATY3275. Induction was for 5 hr. An overview is given in Fig. S9.

In cells which express the nucleolar marker, Sik1p-mRFP, and can be induced to express GFP-Esc1p, systematic examination of through-focal series after 5 hr induction shows that > 90% of escapades contact the perimeter of the nucleus at or immediately adjacent to the nucleolus (Fig. 2A, and see Additional file 5). The nucleolus is not however present within the escapades themselves. The yeast spindle pole body (SPB) is embedded in the NE at a position which is usually opposite the nucleolus [25,26]. Consistent with the observed nucleolar association, the localization of escapades and karmellae does not coincide with the tagged SPB protein, Spc42p (Fig. 2B and see Additional file 6). Systematic counting of through-focal series of cells which exhibit escapades shows that only ~10% of the tagged SPBs contact escapades. Another modification of the NE, the "karmellae" which result from overexpression of HMG-CoA reductase, also associates with the nucleolus [27] and avoids the SPB (Fig. 2C). A third instance of association with the nucleolus is that of the "flares" which appear upon deletion of the ER/NE membrane proteins, Nem1p or Spo7p. In this case, the flares define a pocket which encloses the nucleolus [15].

Since centromeres are close to the SPB during most of the cell cycle [2], we also studied their relation to escapades, using cells which express lac operator arrays integrated near a centromere and GFP-tagged lac repressor. No obvious association is seen between this centromere and escapades (Fig. 2D and see Additional file 7). Equivalent experiments to localize a telomere again show no obvious association (Fig. 2E and see Additional file 8). In both cases, systematic counting of through-focal series of cells which exhibit escapades shows that only ~10–12% of the tagged loci contact escapades. A functional GFP-tagged form of the telomere-associated protein, Rap1p, which binds Esc1p [8], also is not detected outside the chromatin mass, as would be expected if it were in escapades (Fig. 2F and see Additional file 9). This is also the case for Sir4p [8].

### Formation of Escapades-Relation to Cytoplasmic Structures

Following the separation of sister chromatids at the onset of anaphase, the nucleus of the mother cell quickly extends into the bud, generating a dumbbell-shaped structure with a narrow bridge of NE connecting the two nuclei [28]. Fission of the bridge leaves protruding membrane remnants that are normally resorbed by the two resulting nuclei. Escapades superficially resemble these remnants and therefore might be derived from them. Nevertheless, time-lapse microscopy of cells which exit mitosis shows that GFP-Esc1p-positive remnants are efficiently resorbed, as in control cells (see below). Moreover, cells treated with α-factor (3 hr, 1 μg/ml) [See Additional file 10] or hydroxyurea (3 hr, 0.1 M) [See Additional file 11] can generate escapades when Esc1p expression is induced in the absence of cell cycle progression.

The formation of escapades does not appear to depend on the integrity of the tubulin or actin cytoskeleton, judging from experiments in which they are induced in the presence of doses of nocodazole or latrunculin A which effectively depolymerize the corresponding cytoskeletal structures [See Additional files 12 and 13].

**Figure 3:**
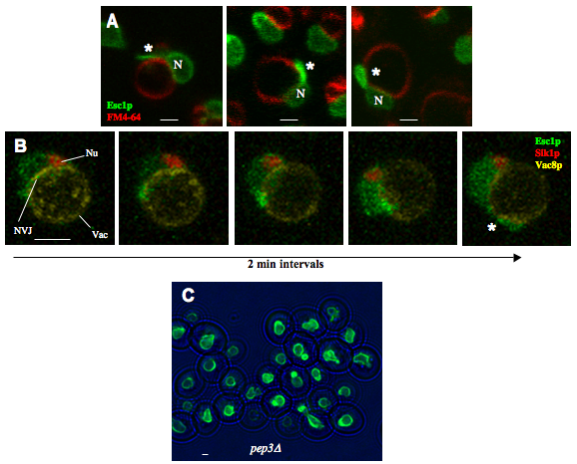
**Escapades Associate with the Vacuole and Originate at the NVJ**. **(A)** Association with the vacuole, as detected by confocal examination of strain ATY2102 stained with FM4-64 (red) after galactose induction for 3 hrs. In each case, the chromatin-containing portion of the nucleus is designated (N) and the escapade is indicated by (*). **(B)** Time-lapse confocal sequence of strain ATY2513 that expresses Sik1p-mRFP (nucleolus: Nu) and Vac8p-YFP, which marks the vacuole membrane (Vac) and concentrates at the nucleus-vacuole junction (NVJ). GFP-Esc1p expression was induced with galactose 30 minutes prior to and maintained throughout time-lapse imaging, to follow the progression of GFP-Esc1p accumulation. Note the GFP-Esc1p at the periphery of the nucleus (N) and its progressive extension along the surface of the vacuole, generating an escapade (*). The *MET25p-VAC8-EYFP *plasmid was from D. Goldfarb. **(C)***pep3Δ *cells which allow induction of GFPEsc1p were induced for 5 hr and examined (ATY2103).

Like the surface of the yeast nucleus [29], escapades are intimately associated with vacuoles, as detected with the membrane dye, FM4-64 (Fig. 3A). Live-cell imaging reveals that escapades can originate at or near the nucleus-vacuole junction (NVJ), prior to extending along the surface of the vacuole (Fig. 3B). Nevertheless, small ring-like escapades and patches of Esc1p can be induced in *pep3-Δ *cells which lack conventional vacuoles [30] (Fig. 3C).

### Inheritance of Escapades

**Figure 4:**
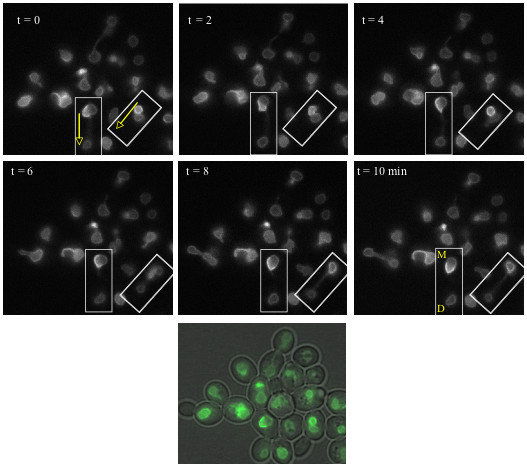
**Escapades are Asymmetrically Inherited**. GAL-GFP-Esc1p strain ATY2102 was induced for 3 hours at 23°C prior to imaging at 2 min intervals in glucose medium. Boxed regions indicate cells undergoing mitosis and the arrows indicate the direction of elongation of the nucleus during anaphase. Mother (M) and daughter (D) nuclei are indicated in the last panel. Note the retention of escapades by the mother. The color image at the bottom includes a phase image to illustrate the cell outlines just prior to t = 0.

The distribution of escapades was examined as cells pass through anaphase. Intriguingly, they are excluded from > 95% of daughter nuclei (Fig. 4/Table 1). The asymmetric distribution is especially conspicuous in a *mob1-77 *mutant which makes it possible to maintain cells in late anaphase for hours (Table 1) [31].

**Table 1:** Maternal Retention of Escapades

Strain	% GFP-Esc1p
MAT a	94.3 +/- 3.7
MAT α	97.1 +/- 2.0
*mob1-77*	98.7 +/- 0.4
*cdc3-3*	94.3 +/- 1.8
*cdc10-1*	94.6 +/- 2.2
*pep3Δ*	93.6 +/- 2.0

The percentage of tagged escapades that remain at the maternal nucleus during mitosis was determined (1) in control strains MATa (ATY2102) and MATα (ATY1550), (2) in the mitotic exit mutant, *mob1-77 *(ATY2502), (3) in septin mutants, *cdc3-3 *(ATY2494) and *cdc10-1 *(ATY2087), and (4) in the absence of an organized vacuole, due to deletion of *PEP3 *(ATY2103). All cells carry the integrated *GAL-GFP-ESC1 *cassette, except for the septin mutants, which were transformed with a centromeric plasmid expressing GFP-Esc1p under control of a methionine-repressible, *MET25*, promoter. Typically, cells were synchronized in G1 (α-factor arrest) during induction for 3 hrs at 23°C and were then allowed to reenter the cell cycle at 23°C under repressing conditions. Ts strains were shifted to the restrictive temperature one hour after α-factor release and examined after 2 hrs. The MATα control strain (ATY1550) was used without synchronization. 100 mitotic cells were examined in at least three replicate experiments for each condition and the distribution of escapades between mother and daughter nuclei was scored. As shown, ~95% of the escapades are always retained in the maternal nucleus in all cases.

### Why are Escapades not Present in Daughter Nuclei ?

Escapades could be actively retained by the maternal nucleus, excluded from the daughter nucleus, or could simply not be able to diffuse in the plane of the membrane. Since septin filaments at the bud neck can restrict transfer of proteins between mother and bud [32-35], we have examined inheritance in strains that carry mutations in septin subunits and disorganize the septin collar at the restrictive temperature (*cdc3-3, cdc10-1*). In these mutants, escapade exclusion from daughter nuclei is again seen (Table 1). Karmellae are also restricted to the mother in wt and in septin mutants (not shown). Consistent with the suggestion that there is no structural impasse at the bud neck, most escapades which remain in the mother do not accumulate at the neck. Moreover, electron microscopic examination shows that a significant space separates the outer nuclear membrane from the inner aspect of the plasma membrane at the bud neck during anaphase [36].

**Figure 5:**
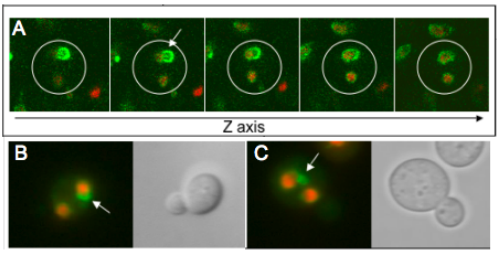
**Distribution of Escapades in Binucleates**. Cells expressing GFP-Esc1p and Htb2p-mRFP (ATY2509) were pre-induced, washed and recultured for 8 hrs at 23°C in glucose medium supplemented with latrunculin A to produce binucleate cells, resulting from occasional incorrect orientation of the spindle. A diploid strain was used to facilitate spatial resolution. **(A) **Confocal z-sections of a binucleate cell show retention of escapades (arrow) in a single nucleus. **(B, C) **Epifluorescent imaging of binucleate cells. Phase images indicate that the nuclei are contained within the maternal cytoplasm.

To evaluate the maternal retention model, we have studied the distribution of escapades in cells for which nuclear division does not require traversal of the bud neck. For this purpose, we have disrupted actin filament integrity with latrunculin A, which allows the spindle to deviate from the mother/bud axis, with the result that nuclear division can occur entirely within the maternal cytoplasm. Strikingly, escapades are restricted to a single nucleus in such binucleates (Fig. 5). This finding led us to investigate whether association with the vacuole could account for maternal retention; however, escapades are overwhelmingly retained in a *pep3Δ *strain (Table 1).

**Figure 6:**
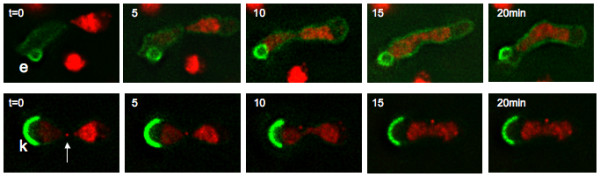
**Distribution of Escapades and Karmellae upon Karyogamy**. Escapades and karmellae remain with the nucleus of their origin upon fusion with nuclei which express Htb2p-mRFP. Top: A strain which allows induction of GFP-Esc1p (ATY1550) was crossed with a strain expressing mRFP-tagged histone Htb2p (ATY2835). Bottom: ATY1650, which carries a galactose-inducible plasmid allowing induction of Hmg1p-Co A Reductase-GFP and expresses a mRFP-tagged form of the SPB-protein, Spc42p (arrow), was crossed with ATY2835. Upon nuclear fusion, the red histone signal gradually invades the *trans *nucleus, but neither the escapades (e) nor karmellae (k) changes its location over at least 20 min. ATY1550 and ATY1650 were pre-grown overnight in galactose medium. For both time series, note that mRFP-tagged chromatin is absent from the regions immediately underlying the escapades or karmellae. These volumes are occupied by the nucleoli. Also, note the infusion of GFP-Esc1p into the *trans *nucleus upon karyogamy, indicative of facile diffusion.

To inquire whether escapades and karmellae are intrinsically immobile, we have examined the constancy of their position when the nucleus with which they associate fuses with a conventional nucleus during karyogamy. As shown in Fig. 6, they show no tendency to migrate into the *trans *nucleus over a period of time greater than that required for anaphase. Nevertheless, tagged nuclear pores are able to access the *trans *nucleus [37,38]. Note the rapid transit of GFP-Esc1p to the *trans *nucleus. At least at this level of expression, it has considerable mobility.

### Functional Consequences

Despite dramatic changes in nuclear shape, cells that express excess Esc1p or GFP-Esc1p are not seriously growth impaired in liquid culture or on solid media. Furthermore, they show a distribution of DNA content comparable to wt, and can mate (not shown and Figure 6). The maternal restriction of escapades may direct most physiological consequences of their presence to the mother and thereby contribute to the lack of a major growth phenotype, as each cell division produces progeny which initially lack escapades.

**Figure 7:**
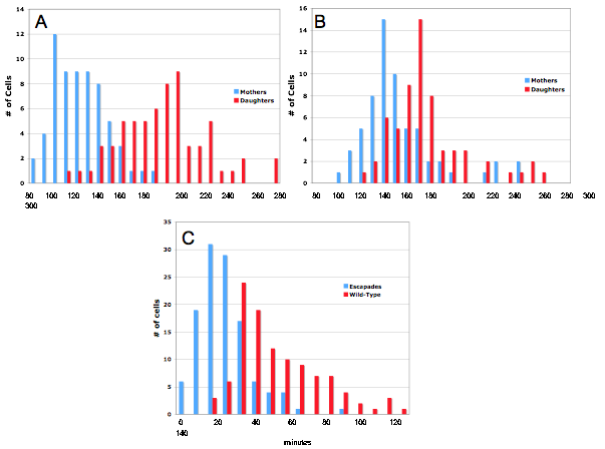
**Kinetics of Bud Formation**. GAL-GFP-Esc1p strain ATY2102 and an isogenic wild-type strain (ATY2500) were pre-induced in galactose media for 3 hrs, washed and examined on agarose pads in glucose media over 5 hr, during which time escapades remained visible (ATY2500). DIC images captured every 10 min allowed measurement of the timing of bud initiation for each cell. Time measurements were all relative to the emergence of the first bud by each mother cell, M1. For example, the mother's bud interval, M2-M1 measures the delay prior to the appearance of the second bud on the mother. Correspondingly, D-M1 measures the interval prior to the appearance of the first bud on the daughter. **(A) **Histogram showing the bud intervals for a single experiment for the wild type strain. Note that there is minimal overlap between mothers and daughters and that mothers re-bud considerably faster than daughter cells. **(B) **Histogram showing the bud intervals for an isogenic escapade strain. Note, in contrast to (**A**), that there is considerable overlap between mothers and daughters. The bud interval is slowed in mothers and accelerated in daughters. **(C) **Histogram illustrating the mother-daughter bud differential calculated as: (D-M2 = bud differential) where D is the time at which a bud appears in the daughter cell and M2 is the time at which a second bud appears in the mother cell. Note that the bud differential is decreased by 50% by the presence of escapades (average bud differential is 65 min. in wild-type strain and 28 min. in escapade strain). Results are cumulative over four separate experiments for each strain.

It is therefore of interest that a differential effect at the level of cell cycle progression is evident. Normally, when a mother cell gives rise to a daughter, the mother will rebud quickly, while daughters must grow to a critical size before budding [39]. By contrast, when pre-induced GFP-Esc1p-expressing cells are observed, the bud interval is slowed in mothers (possibly due to interference by the escapades themselves) and accelerated in daughters. As a result, the difference in timing of budding between mothers and daughters is reduced by more than 50% (Fig. 7). The net impact of these events could account for the modest reduction of relative growth rate which can best be detected in mixed cultures in which isogenic wt and GFP-Esc1p-expressing cells are both present. Pure cultures of cells which can be induced to express GFP-Esc1p remain fluoresecent for days of culture in galactose medium, while addition of an equal number of wild type cells prior to culture is followed by a gradual and progressive reduction in the proportion of fluorescent cells.

To further investigate the consequences of excess Esc1p, we initiated microarray analysis of poly(A)+ mRNAs of a pair of isogenic strains grown in galactose medium, only one of which allows Esc1p induction from a *GAL1 *promoter (Table 2). Upon overnight growth in galactose medium, there is a ~20–30x increase in the signal corresponding to *ESC1 *mRNA and only 48 other changes which exceed 1.8x fold. Both positive and negative changes are seen. Considering that GFP-Esc1p concentrates at the extreme periphery of the nucleoplasm along with telomeres, it is of interest that both the increases and decreases (> 1.8 fold) are enriched within 40 kb of telomeres, i.e. even though this region accounts for only ~4.1% of the length of the average yeast chromosome, it accounts for ~30% of these changes. Nevertheless, ~70% of the changes are not in this region. No notable changes in mRNA levels were seen for genes whose products associate with Esc1p, such as *RAP1 *and *SIR4*. Since deletion of *ESC1 *does not obviously affect nuclear morphology [See Additional file 14], we have not investigated corresponding transcriptional changes.

**Table 2:** Microarray Data

Fold Change	Gene Name	kb from Telomere	Chromosome
3.37	HMS2	42	X
3.20	NAT5	408	XIII
2.93	CDA1	333	XII
2.89	SRT1	136	XIII
2.62	CIT3	392	XVI
2.57	SSP1	90	VIII
2.47	SEO1	7	I
2.47	IMD2	7	VIII
2.43	THI12	15	XIV
2.35	UFO1	92	XIII
2.26	TY1	357	XIII
2.24	TY2	133	XII
2.21	KCC4	79	III
2.19	IMD3	76	XII
2.17	TY1	357	XIII
2.12	TY5	3	III
2.12	Putative ORF	10	III
2.06	Putative ORF	214	II
1.99	SPO1	175	XIV
1.97	BNA2	150	X
1.97	TY2	129	XII
1.94	DAK2	23	VI
1.94	TY2	29	II
1.92	TY2	132	XII
1.92	STE3	113	XI
1.91	TY2	381	XV
1.91	TY2	129	XII
1.90	SRD1	148	III
1.85	TY2	129	XII
-19.96	YGR035C	560	VII
-11.04	ZRT1	21	VII
-8.21	FDH2	18	XVI
-3.89	PHO84	25	XIII
-3.35	PMA2	482	XVI
-3.25	CTR1	140	XVI
-2.77	SPA2	101	XII
-2.27	HMS1	390	XV
-2.27	MET2	117	XIV
-2.17	SNF7	194	XII
-2.09	ZRT2	401	XII
-2.06	FRE7	41	XV
-1.95	STL1	9	IV
-1.93	RIM4	51	VIII
-1.91	AQY1	10	XVI
-1.88	YOR1	20	VII
-1.87	FRE1	568	XII
-1.81	SPS100	170	VIII

Comparison of GFP-Esc1p cells which were induced overnight and control cells grown in galactose medium. The list includes those genes whose microarray signals change by at least 1.8x. Interestingly, only two of the entries in this Table are among the 100 loci identified as putative Esc1p-binding sites in a genome-wide screen [65].

## Discussion

The morphology of organelles is intimately related to their function and deviation from normal morphology can have profound physiological consequences. It is thus plausible that NE shape alterations contribute to events which cause cellular malfunction in disease.

Nevertheless, radical changes of the composition of the nuclear periphery can be compatible with cell survival, as in a variety of yeast deletion strains lacking transmembrane proteins which localize to the NE and ER (Nem1p, Spo7p, Ssh1p), nucleoplasmic proteins (Thp1p), or nucleoporins (Seh1p) [14,17]. None of these proteins normally are restricted to the periphery of the nucleoplasm itself.

Interestingly, the shape of the nucleus is not affected by deletion of Esc1p or deletion of two other proteins which normally localize to the periphery of the nucleoplasm, Mlp1p and Mlp2p [9,22,40]. Moreover, the growth of *nem1-Δ, seh1-Δ, spo7-Δ, ssh1-Δ *and *thp1-Δ *[14] is not obviously affected by overexpression of Esc1p (not shown).

Mutations in nuclear lamina constituents, most notably lamin A and C, cause a diverse spectrum of diseases, the laminopathies. Laminopathies caused by excess pre-lamin A at the nuclear periphery are characterized by bleb-like expansions of the nuclear surface [41-44]. The dependence of the shape of the yeast nucleus on both nuclear membrane proteins and proteins that concentrate at the periphery of the nucleoplasm is reminiscent of a distinct laminopathy (Emery-Dreyfuss Muscular Dystrophy), which can result from either mutation of the inner nuclear membrane protein, emerin, or mutation of lamin A [45,46]. Moreover, overexpression of a GFP-tagged form of the inner membrane protein, Prm3p, distorts the shape of the NE in much the same fashion as Esc1p, and excess Heh1p and Heh2p [24] grossly distort the NE and chromatin mass, while Mlp1p induction has no obvious impact. These differential effects may signify that the Heh proteins have a high affinity for chromatin, that Esc1p and Prm3p are more closely linked to the inner nuclear membrane *per se *than to chromatin, and that Mlp1p is relatively independent.

**Figure 8:**
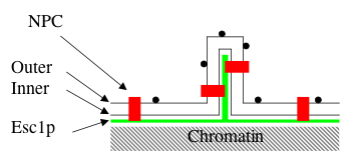
**Model of Escapade Structure**. Nuclear pore complexes (NPC), outer nuclear membrane (Outer), inner nuclear membrane (Inner), and Esc1p are indicated. The small circles at the surface of the membranes are ribosomes. The model has been drawn to illustrate a tight apposition of Esc1p layers within the escapade, which may account for the constant width that is observed with EM and the exclusion of chromatin.

The characteristic structure of escapades and distribution of excess GFP-Esc1p are compatible with the hypothesis that excess Esc1p forces enlargement of the NE due to end-to-end association of the protein, that chromatin has an intrinsic coherence which tends to preserve a roughly globular shape, and that the inner aspect of the NE (or perhaps Esc1p itself) can also self-associate laterally (Fig. 8). Indeed, although there has never been an experimentally accessible model for investigation of this latter issue, the NE of malignant cells – like escapades – is frequently characterized by focal self-apposition of the lamina and/or the nucleoplasmic surface of the inner nuclear membrane [47,48].

There is no reason to expect that Esc1p is fully comparable to higher eukaryotic lamins. For example, unlike lamins, tagged Esc1p (or Mlp1p) expressed from its own promoter does not completely encircle the nucleus, being absent from beneath the nucleolus [13,40]. Moreover, the observation of rapid diffusion of GFP-Esc1p upon karyogamy shows that at least the overexpressed protein is mobile.

The transcriptional consequences of overexpressing Esc1p emphasize the importance of this protein (or escapades themselves) for gene expression. Since both negative and positive changes are seen, Esc1p appears to be a complex regulator, not only an enhancer of silencing. In this regard, it resembles many transcriptional regulators including Rap1p [49]. It is also notable that ~70% of the changes which we detect occur at loci which are further than 40 kb from telomeres (which concentrate at the periphery). In addition to their intrinsic interest, these microarray data provide a possible prototype against which to evaluate the transcriptional characteristics of laminopathies, which presumably account for their cell type-specific effects.

Daughter cells differ from mothers in several regards [50,51]. Escapades are not transferred to daughter cells, apparently due to their immobility. These structures – and karmellae – are thus part of a "lagging" domain of the nuclear perimeter. The nucleolus may also be part of this domain, judging from its association with escapades and karmellae, as well as the observation that it is one of the last nuclear components to reach the bud during anaphase [52,53]. Extra rDNA circles [54] and ARS plasmids [55] are also retained.

## Conclusion

This study demonstrates the extreme structural plasticity of the yeast nucleus and shows that the unusual "escapades" which can be generated are essentially immobile and therefore are not inherited. Their lack of inheritance provides a striking example of the exclusion of epigenetic change. Such mechanisms – coupled with equivalent normalization of phenotype at the molecular level – are likely to sustain cell identity through mitosis. Higher eukaryotic cells may cope with such issues by extensive disassembly and reassembly of the NE during mitosis, which could provide an opportunity to avoid incorporation of structurally aberrant components.

## Methods

### Yeast Strains and Plasmids

Yeast strains and plasmids used in this study are listed in Table 3 and Table 4. The GFP-tagged form of Esc1p which we employ is known to complement the plasmid partitioning defect of *esc1Δ *[8]. Strains were constructed using standard methods for transformation, mating, and sporulation. To place *ESC1 *transcription under control of a galactose-inducible promoter, we used the plasmid, pFA6A-pGAL1 [56], as template to generate PCR products which could be targeted upstream of *ESC1*. Plasmid p*MET25-GFP-ESC1 *was constructed by PCR sub-cloning of the full length Esc1p coding sequence from plasmid pEDA129 [8], into pGFP-N-FUS [57]. Restriction endonuclease sites, SpeI and XhoI, present in the polylinker, were utilized such that the *ESC1 *ORF is in-frame down-stream of GFP and expression is driven by the methionine-repressible *MET25 *promoter and terminated by the *CYC1 *terminator. Recombinant clones were identified by restriction endonuclease analysis and DNA sequencing of the 5' cloning junction to verify orientation and reading frame. The level of expression observed in transformed strains cultured under inducing conditions was sufficient to generate NE alterations.

**Table 3:** Strains Used in this Study

**Strain Number**	**Description, Relevant Genotype**	**Derived From**	**Reference**
ATY1483	W303 ESC1/GALp-GFP-ESC1::his5+	= YDZ49	[8]
ATY1550	MATα GALp-GFP-ESC1::his5+	YDZ49	[8]
ATY1577	SIK1/SIK1-mRFP::kanMX6 SPC42-GFP/SPC42 [pGALp-HMG1-GFP]	IAY18 × SIK1-mRFP*	[60–62]
ATY1650	MATα SPC42-mRFP [pGALp-HMG1-GFP]		[61, 62]
ATY2087	MATa *cdc10-1 *[pMET25-GFP-ESC1]	E. Bi (#741)	
ATY2096	MATα SPC42-mRFP::kanMX6/SPC42 GALp-GFP-ESC1::his5+/ESC1	ATY2102 × SPC42-mRFP*	[62]
ATY2097	W303 ESC1/GALp-GFP-ESC1:: HIS3 lacO TELXIVL his3-11-15:: HISp-GFP-lacI-HIS3 *his3-11-15*:: HISp-GFP-lacI-HIS3	GA1985 × ATY1550	[8, 63]
ATY2098	W303 ESC1/GALp-GFP-ESC1::his5+ lacO CENIV *his3-11-15*::HISp-GFP-lacI-HIS3	GA1321 × ATY1550	[8, 63]
ATY2101	MATα GALp-GFP-ESC1::his5+ SIK1-mRFP::kanMX6	ATY2102 × SIK1-mRFP	[8, 62]
ATY2102	MATa GALp-GFP-ESC1::his5+	YDZ49	[8]
ATY2103	MATa *pep3Δ *::TRP1 GALp-GFP-ESC1::his5+	ATY2101 × BJ1601	[30]
ATY2110	MATα W303R		
ATY2163	MATa *cdc3-3 *[pGALp-HMG1-GFP]	E. Bi (#739)	[61]
ATY2164	MATa *cdc10-1 *[pGALp-HMG1-GFP]	E. Bi (#741)	[61]
ATY2289	MAT α HTB2-mRFP::kanMX6	W303R α	
ATY2494	MATa *cdc3-3 *[pMET25-GFP-ESC1]	E. Bi (#739)	
ATY2500	W303a	YDZ49	[8]
ATY2501	MATa W303 *his3*	YDZ49	[8]
ATY2502	MATa *mob1-77 *GALp-GFP-ESC1::his5+/ESC1	FLY30/198 × ATY1550	[64]
ATY2509	GALp-GFP-ESC1::his5+/ESC1 HTB2-mRFP::kanMX6/HTB2	ATY2835, ATY1550	
ATY2513	ESC1/GALp-GFP-ESC1::his5+ SIK1/SIK1-mRFP::kanMX6 [pMET25p-VAC8-YFP]	ATY2102 × SIK1-mRFP*	[8, 62]
ATY2835	MATa HTB2-mRFP::kanMX6	W303Ra	
ATY2957	MATa *ura3-52*::hmg1-GFP:URA3 GALp-ESC1::kanMX6	SFN1163	[59]
ATY3138	MATa RAP1-GFP HTB2-mRFP::kanMX6	YG841	
ATY3156	MAT α GAL-ESC1 HTB2-mRFP::kanMX6 [pNUP49-GFP]	ATY2957 × ATY2289	
ATY3244	MATa HTB2-mRFP::kanMX6 [pGAL-GFP-HEH1-YFP] HTB2-mRFP	ATY2835	
ATY3245	MATa HTB2-mRFP::kanMX6 [pGAL-GFP-HEH2-YFP] HTB2-mRFP	ATY2835	
ATY3246	MATa HTB2-mRFP::kanMX6 [pMET25-GFP-PRM3]	ATY2835	
ATY3255	SEC61-GFP [pGAL-MLP1]	SFN1056	[59]
ATY3256	MATa HTB2-mRFP::kanMX6 [pGAL-MLP1]	ATY2835	
ATY3258	MATa GALp-GFP-ESC1::his5+ SIK1-mRFP::kanMX6	ATY2835, ATY1513	
ATY3275	MATa GALp-ESC1 RAP1-GFP HTB2-mRFP::kanMX6	ATY1676, ATY3007	
ATY3276	MATa HTB2-mRFP::kanMX6 SPC42-mRFP::kanMX6	ATY2102, SPC42-mRFP*	
ATY3278	ESC1::HIS5 [pSEC63-GFP]	YDZ13	[8]
ATY3281	MATa GALp-GFP-ESC1::his5+ HTB2-mRFP::kanMX6	ATY2835, ATY1550	

• Unnumbered MAT alpha reference strains expressing Sik1p-mRFP or Spc42p-mRFP were obtained from W-K. Huh and E. O'Shea.

**Table 4:** Plasmids Used in this Study

Plasmid Number	Name	Original Name	Source/Reference
AT635	pNUP49-GFP	pUN100-GFP-Nup49, LEU2/CEN	V. Doye
AT969	pGAL-MLP1	pGAL-MLP1, CEN/URA3	M. Rout
AT970	pGAL-GFP-HMG	CR425, CEN/URA3	R. Wright, D. Meyer
AT993	pMET25-VAC8-EYFP	CEN/URA3	D. Goldfarb
AT1038	pSEC63-GFP	pJK59, CEN/URA3	T. Rapoport
AT1143	pMET25-PRM3	YCPyeGFP-PRM3, CEN/TRP1	T. Lithgow
AT1183	pGAL-HEH1-YFP	pMKPL1, 2μ/TRP1	P. Lusk, G. Blobel
AT1184	pGAL-HEH2-YFP	p MKPL2, 2 μ/TRP1	P. Lusk, G. Blobel

### Media and Supplements

Cells were grown in complete synthetic medium or the appropriate dropout medium supplemented with 2% D-glucose, D-raffinose or D-galactose. Generally strains were maintained in mid-log phase in raffinose medium and were induced by addition of galactose. All incubations were at room temperature except when ts conditional strains needed to be shifted to 37°C. As required, media were supplemented with: 200 μM latrunculin A (Sigma) diluted from a 20 mM stock in DMSO, 5 μg/ml α-factor (Sigma) diluted from a 5 mg/ml stock in sterile water, 0.1 M hydroxyurea (Sigma) diluted from a 1 M stock in sterile water, 15 μg/ml nocodazole (Sigma) diluted from a 3 mg/ml stock in DMSO, or 40 μM FM4-64 (Molecular Probes) diluted from a 2 mM stock in DMSO.

### Microarrays

GAL-GFP-Esc1p strain ATY2102 and isogenic wild-type ATY2501 were grown overnight to mid-log phase in galactose-containing synthetic medium at room temperature. Total RNA was purified from duplicate 1 ml samples by mechanical disruption with 0.2 micron glass beads in an equal volume of hot acid phenol and used for biotinylated cRNA synthesis according to Affymetrix protocols at the Case Cancer Center Gene Expression Array Facility. Replicate samples were hybridized to Affymetrix GeneChip yeast genome Y98 arrays. Scanned chip images were analyzed with the Affymetrix GeneChip Operating Software (GCOS) using the MAS 5.1 algorithm for fold-change calculations. The results were tabulated with Microsoft Access and annotated using NETAFFX (Affymetrix) and Genespring (Silicon Genetics) software packages. In the tabulation in Table 2, the average values for GFP-Esc1p-overexpressing cells are divided by the averaged data for control cells.

### Budding Kinetics

GAL-GFP-Esc1p strain ATY2102 and isogenic wild-type strain ATY2501 were pre-induced in galactose media for 3 hrs at 23°C, washed and examined on agarose pads in glucose medium over 5 hr, during which time fluorescent escapades remained visible. DIC images captured every 10 min allowed measurement of the timing of bud initiation for each cell in four replicate experiments. Time measurements were all relative to the emergence of the first bud by each mother cell, M_1_. For example, the mother's bud interval, M_2_-M_1 _measures the delay prior to the appearance of the second bud on the mother. Correspondingly, D-M_1 _measures the interval prior to the appearance of the first bud on the daughter.

The time difference of budding between mothers and daughters is referred to as the budding differential and is calculated as: (D-M_2 _= bud differential) where D is the time at which a bud appears in the daughter cell and M_2 _is the time at which a second bud appears in the mother cell.

### Microscopy

Epifluorescent, phase time-lapse, and DIC microscopy were performed on a Leica DMLB microscope equipped with a SPOT camera (Diagnostic Instruments Inc.) and the SPOT Advanced software package. Confocal microscopy for live cell Z-stack and time-lapse imaging were performed on either a Zeiss LSM510 or a Leica AOBS. Volocity (Improvision) and Metamorph (Molecular Devises) software packages were utilized for image analysis and generation of time-lapse movie sequences. Other z-stack images were collected with a DeltaVision microscope and deconvolved before inspection. For quantitation of association of escapades with various structures, through-focal series were examined systematically and the distribution of nucleoli, etc. was counted in greater than 100 cells.

Vacuole membrane staining with the vital dye FM4-64 was conducted according to [58] with the following modifications. Briefly, log phase galactose-induced cultures were concentrated by centrifugation and resuspended at OD_600 _= 1 in glucose-containing medium supplemented with 40 μM FM4-64. The cells were then incubated at 30°C with shaking for 30–60 minutes, followed by extensive washing to remove excess dye. Stained vacuoles were imaged at 546 nm with a Zeiss LSM510 confocal microscope.

For time-lapse microscopy, dilute cultures were briefly centrifuged and 2 μl aliquots from the pellet were applied to the middle of 1.5% Agarose pads prepared on microscope slides in glucose- or galactose-containing culture medium, as appropriate. Samples were overlayed with a coverslip and sealed with vaseline before examination.

To learn whether escapades or karmellae are free to move in the plane of the NE, we have crossed cells which express these structures with cells of the opposite mating type and then observed the structure of the nucleus during and after nuclear fusion. For this purpose, pairs of cells grown overnight in galactose-containing medium were mixed, sedimented, and applied to an agarose pad in glucose-containing complete synthetic medium (as above). After approximately 2 hrs, they were observed.

Transmission electron microscopy was performed on samples prepared from cultures grown to mid-log phase at 30°C. Samples were fixed in 2% glutaraldehyde followed by secondary fixation/staining in 4% potassium permanganate and uranyl acetate *en bloc*. Samples were dehydrated, embedded in Spurr resin, stained with lead citrate and uranyl acetate and examined in a JEOL 1200CX EM.

## Authors' contributions

TH conducted most of the experiments as part of his doctoral work.

EA, who made the first observations of deformation of the nuclear envelope/nucleus upon Esc1p induction, provided frequent advice.

AT made the initial observations on escapade induction in this laboratory, coordinated the study, and wrote most of the manuscript.

All authors read and approved the text.

## Supplementary Material

Additional file 1 Impact of excess Mlp1p. Cells expressing the ER/NE membrane protein, Sec61p-GFP (ATY3255), or Htb2p-mRFP (ATY3256) were induced to overexpress Mlp1p for 5 hr by addition of galactose. Note the conventional distribution of GFP signal at the NE and throughout the peripheral ER (left panel) as well as the roughly spherical chromatin mass (right panel). Click here for file

Additional file 2 Impact of excess Prm3p. Htb2p-mRFP-expressing cells were induced to overexpress GFP-Prm3p for 5 hr by transfer to methionine-free medium (ATY3244). Note the appearance of GFP-positive ring-like structures and a fin at the margin of the nucleus. Click here for file

Additional file 3 Impact of excess Heh1. Htb2p-mRFP-expressing cells were induced galactose to overexpress Heh1p-GFP for 5 hr by addition of 2% (ATY3245). The red and green images have been separated for clarity. Note the massive change of organization of both chromatin and the GFP signal, which often encircles the chromatin. Click here for file

Additional file 4 Impact of excess Heh2p. Htb2p-mRFP-expressing cells were induced to overexpress Heh2p-GFP for 5 hr by addition of 2% galactose (ATY3246). The red and green images have been separated for clarity. Note the massive change of organization of both chromatin and the GFP signal, which generally encircles the chromatin. Click here for file

Additional file 5 Spatial relation of escapades to the nucleolus. Cells expressing the nucleolar marker, Sik1p-mRFP, were induced to express GFP-Esc1p for 5 hr by addition of 2% galactose (ATY3258). Systematic examination of through-focal series detects association of escapades and Sik1p-mRFP in > 90% of cells which have escapades. Nevertheless, in a given section the extent of association often appears only modest. Click here for file

Additional file 6 Spatial relation of escapades to the spindle pole body. Overview comparison of the localization of GFP-tagged Esc1p and the spindle pole body, in a strain (ATY3276) which expresses Spc42p-mRFP and has been induced by addition of 2% galactose for 5 hr. Systematic examination of through-focal series detects association in ~10% of cells which have escapades. Click here for file

Additional file 7 Spatial relation of escapades to a centromere. Overview comparison of the localization of GFP-tagged Esc1p and a centromere, in a strain (ATY2098) which expresses a GFP-lac repressor fusion, an insertion of lac operator arrays near CENIV and Nup49p-GFP. It has been induced by addition of 2% galactose for 5 hr. Systematic examination of through-focal series detects association in ~10% of cells which have escapades. Click here for file

Additional file 8 Spatial relation of escapades to a telomere. Overview comparison of the localization of GFP-tagged escapades and a telomere, in a strain (ATY2097) which expresses a GFP-lac repressor fusion, an insertion of lac operator arrays near telomere XIVL and Nup49p-GFP. It has been induced by addition of 2% galactose for 5 hr. Systematic examination of through-focal series detects association in ~10% of cells which have escapades. Click here for file

Additional file 9 Spatial relation of escapades to Rap1p. Overview localization of Rap 1-GFP in a strain (ATY3275) which expresses Htb2p-mRFP and allows galactose induction of untagged Esc1p. It was induced by addition of 2% galactose for 5 hr. Note that the labeled foci often are at the periphery of the chromatin mass, but – unlike escapades – do not extend centrifugally toward the cytoplasm. Click here for file

Additional file 10 Induction of escapades in cells treated with mating factor. GFP-Esc1p was induced by addition of 2% galactose for 5 hr in cells which had already been treated with 5 μg/ml α-factor for 2 hr (ATY2101). Note that the appearance of escapades is comparable to that illustrated in Figure 1A. Click here for file

Additional file 11 Induction of escapades in cells treated with hydroxyurea. GFP-Esc1p was induced by addition of 2% galactose for 5 hr in cells which had already been treated with 0.1 M hydroxyurea for 2 hr (ATY2101). Note that the appearance of escapades is comparable to that illustrated in Figure 1A. Click here for file

Additional file 12 Induction of escapades in cells treated with nocodazole. GFP-Esc1p was induced by addition of 2% galactose for 5 hr in cells which had already been treated with 15 μg/ml nocodazole 2 hr (ATY2101). Note that the appearance of escapades is comparable to that illustrated in Figure 1A. Click here for file

Additional file 13 Induction of escapades in cells treated with latrunculin A. GFP-Esc1p was induced by addition of 2% galactose for 5 hr in cells which had already been treated with 200 μg/ml latrunculin A 2 hr (ATY2101). Note that the appearance of escapades is comparable to that illustrated in Figure 1A. Click here for file

Additional file 14 Nuclear shape in the absence of Esc1p. The contour of the NE was defined in cells which lack Esc1p by monitoring the distribution of Sec63p-GFP in a corresponding deletion strain (ATY3278). Click here for file
