# Supplementary figures and images for: Immobility, inheritance and plasticity of shape of the yeast nucleus
Source: BMC Cell Biol. 2007 Nov 9;8:47. doi: 10.1186/1471-2121-8-47 (PMC2222239; doi:10.1186/1471-2121-8-47)

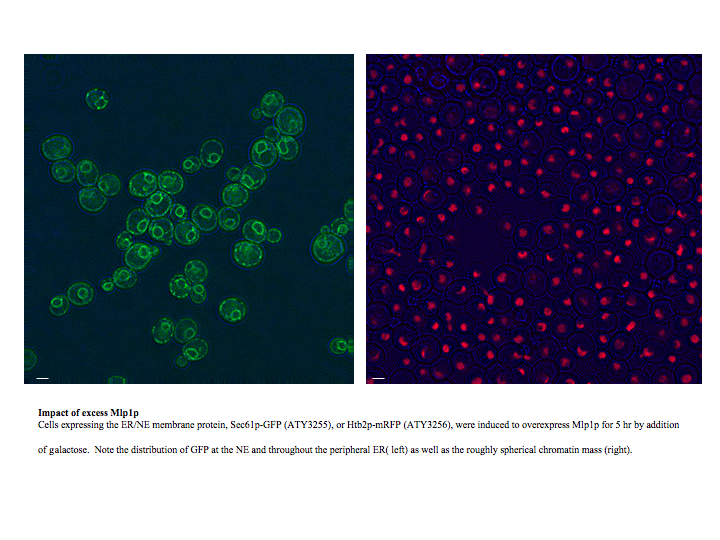

Supplement: Additional file 1 — Impact of excess Mlp1p. Cells expressing the ER/NE membrane protein, Sec61p-GFP (ATY3255), or Htb2p-mRFP (ATY3256) were induced to overexpress Mlp1p for 5 hr by addition of galactose. Note the conventional distribution of GFP signal at the NE and throughout the peripheral ER (left panel) as well as the roughly spherical chromatin mass (right panel). [file 1471-2121-8-47-S1.tiff]

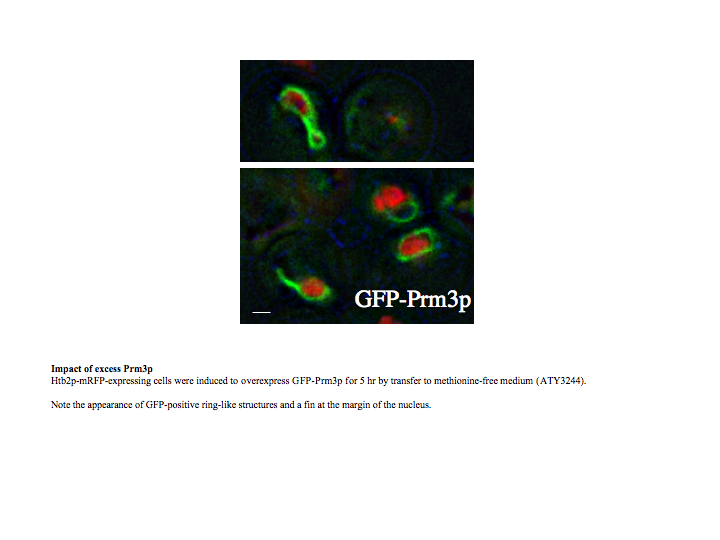

Supplement: Additional file 2 — Impact of excess Prm3p. Htb2p-mRFP-expressing cells were induced to overexpress GFP-Prm3p for 5 hr by transfer to methionine-free medium (ATY3244). Note the appearance of GFP-positive ring-like structures and a fin at the margin of the nucleus. [file 1471-2121-8-47-S2.tiff]

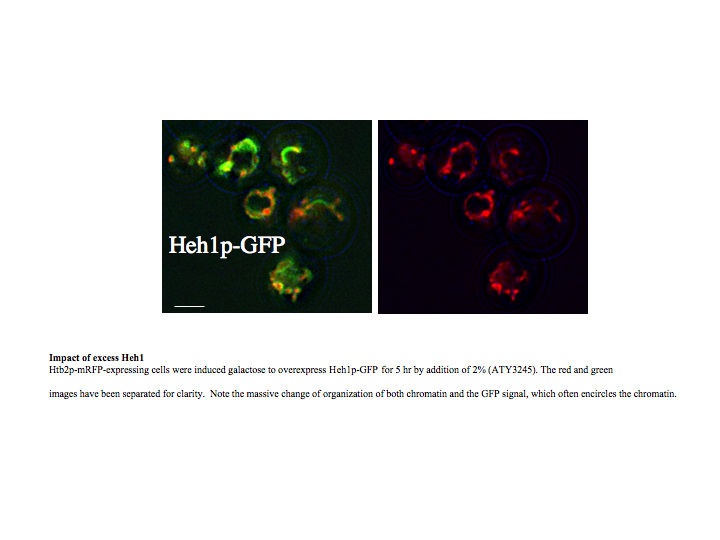

Supplement: Additional file 3 — Impact of excess Heh1. Htb2p-mRFP-expressing cells were induced galactose to overexpress Heh1p-GFP for 5 hr by addition of 2% (ATY3245). The red and green images have been separated for clarity. Note the massive change of organization of both chromatin and the GFP signal, which often encircles the chromatin. [file 1471-2121-8-47-S3.tiff]

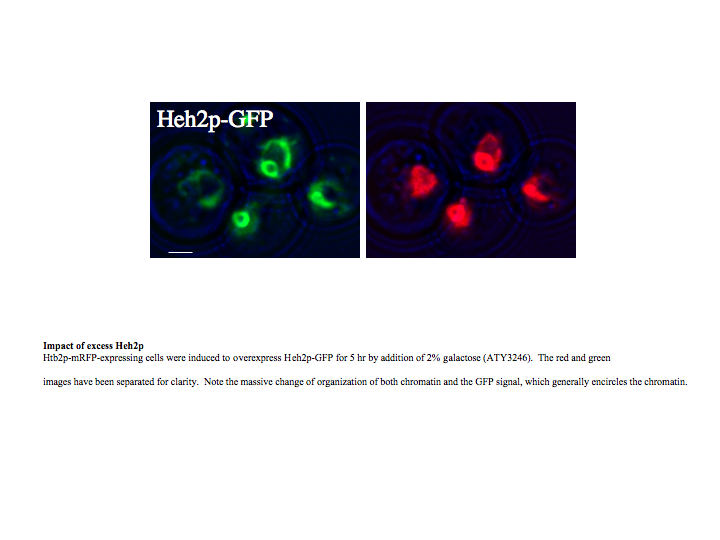

Supplement: Additional file 4 — Impact of excess Heh2p. Htb2p-mRFP-expressing cells were induced to overexpress Heh2p-GFP for 5 hr by addition of 2% galactose (ATY3246). The red and green images have been separated for clarity. Note the massive change of organization of both chromatin and the GFP signal, which generally encircles the chromatin. [file 1471-2121-8-47-S4.tiff]

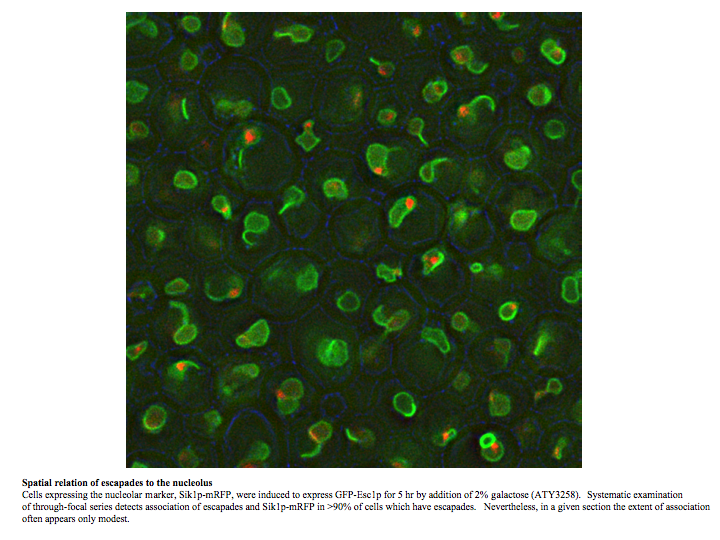

Supplement: Additional file 5 — Spatial relation of escapades to the nucleolus. Cells expressing the nucleolar marker, Sik1p-mRFP, were induced to express GFP-Esc1p for 5 hr by addition of 2% galactose (ATY3258). Systematic examination of through-focal series detects association of escapades and Sik1p-mRFP in > 90% of cells which have escapades. Nevertheless, in a given section the extent of association often appears only modest. [file 1471-2121-8-47-S5.tiff]

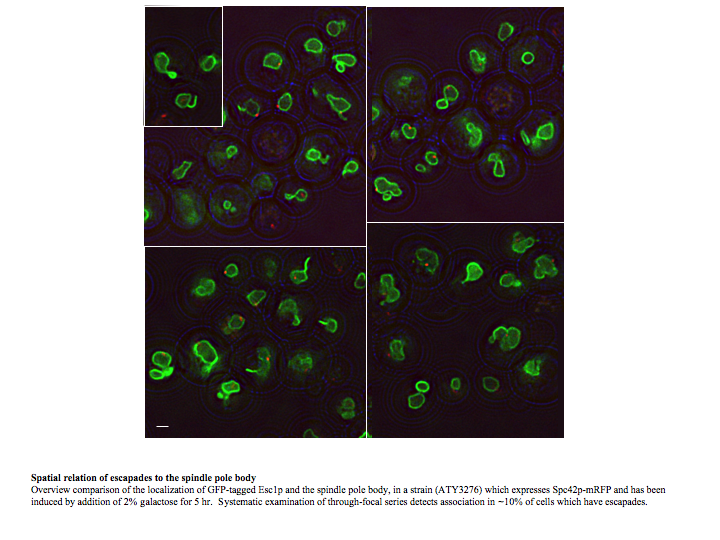

Supplement: Additional file 6 — Spatial relation of escapades to the spindle pole body. Overview comparison of the localization of GFP-tagged Esc1p and the spindle pole body, in a strain (ATY3276) which expresses Spc42p-mRFP and has been induced by addition of 2% galactose for 5 hr. Systematic examination of through-focal series detects association in ~10% of cells which have escapades. [file 1471-2121-8-47-S6.tiff]

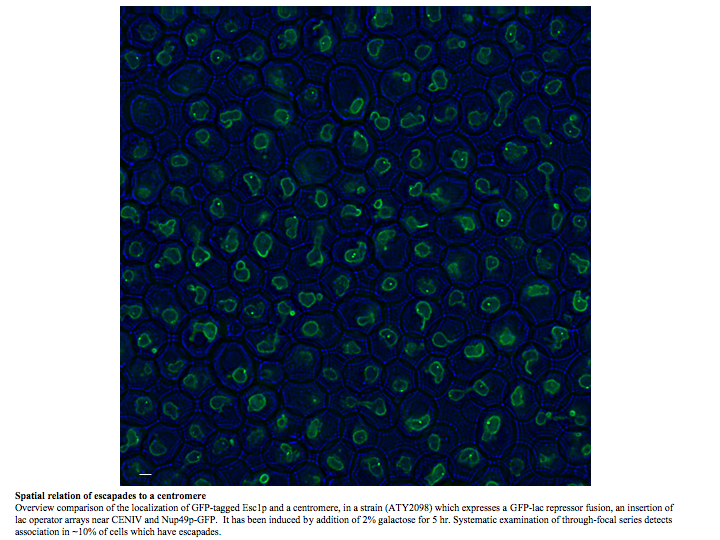

Supplement: Additional file 7 — Spatial relation of escapades to a centromere. Overview comparison of the localization of GFP-tagged Esc1p and a centromere, in a strain (ATY2098) which expresses a GFP-lac repressor fusion, an insertion of lac operator arrays near CENIV and Nup49p-GFP. It has been induced by addition of 2% galactose for 5 hr. Systematic examination of through-focal series detects association in ~10% of cells which have escapades. [file 1471-2121-8-47-S7.tiff]

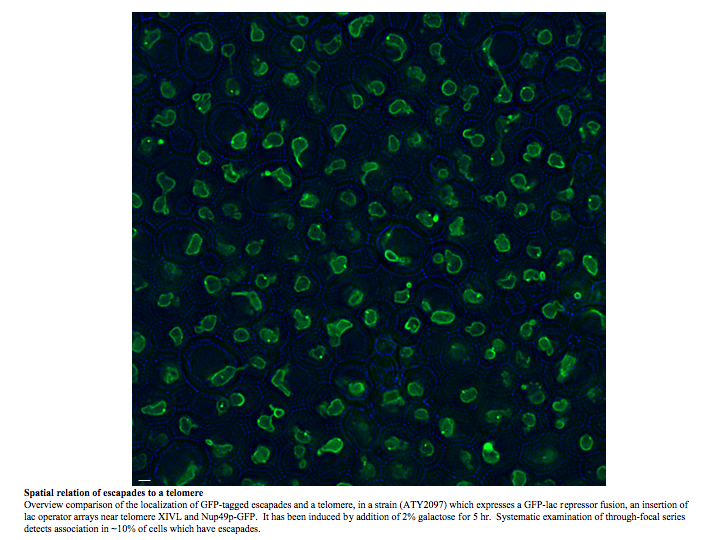

Supplement: Additional file 8 — Spatial relation of escapades to a telomere. Overview comparison of the localization of GFP-tagged escapades and a telomere, in a strain (ATY2097) which expresses a GFP-lac repressor fusion, an insertion of lac operator arrays near telomere XIVL and Nup49p-GFP. It has been induced by addition of 2% galactose for 5 hr. Systematic examination of through-focal series detects association in ~10% of cells which have escapades. [file 1471-2121-8-47-S8.tiff]

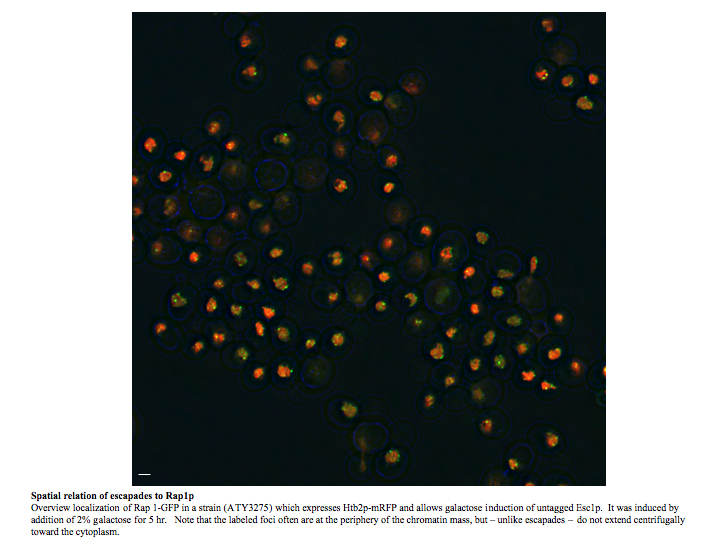

Supplement: Additional file 9 — Spatial relation of escapades to Rap1p. Overview localization of Rap 1-GFP in a strain (ATY3275) which expresses Htb2p-mRFP and allows galactose induction of untagged Esc1p. It was induced by addition of 2% galactose for 5 hr. Note that the labeled foci often are at the periphery of the chromatin mass, but – unlike escapades – do not extend centrifugally toward the cytoplasm. [file 1471-2121-8-47-S9.tiff]

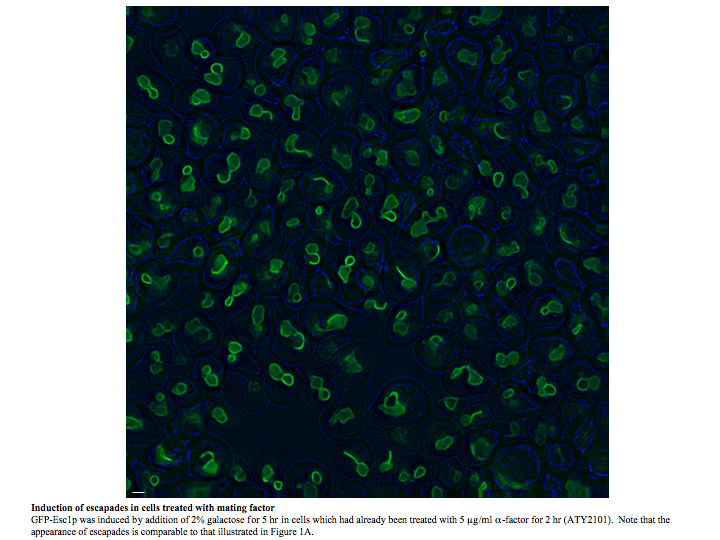

Supplement: Additional file 10 — Induction of escapades in cells treated with mating factor. GFP-Esc1p was induced by addition of 2% galactose for 5 hr in cells which had already been treated with 5 μg/ml α-factor for 2 hr (ATY2101). Note that the appearance of escapades is comparable to that illustrated in Figure 1A. [file 1471-2121-8-47-S10.tiff]

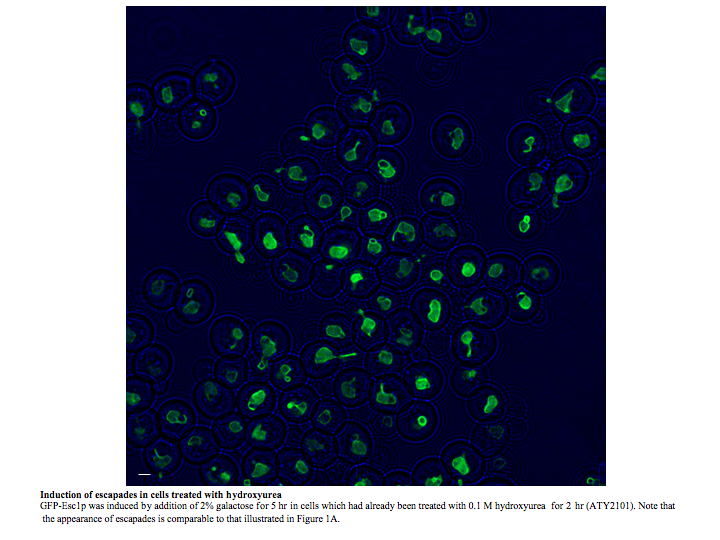

Supplement: Additional file 11 — Induction of escapades in cells treated with hydroxyurea. GFP-Esc1p was induced by addition of 2% galactose for 5 hr in cells which had already been treated with 0.1 M hydroxyurea for 2 hr (ATY2101). Note that the appearance of escapades is comparable to that illustrated in Figure 1A. [file 1471-2121-8-47-S11.tiff]

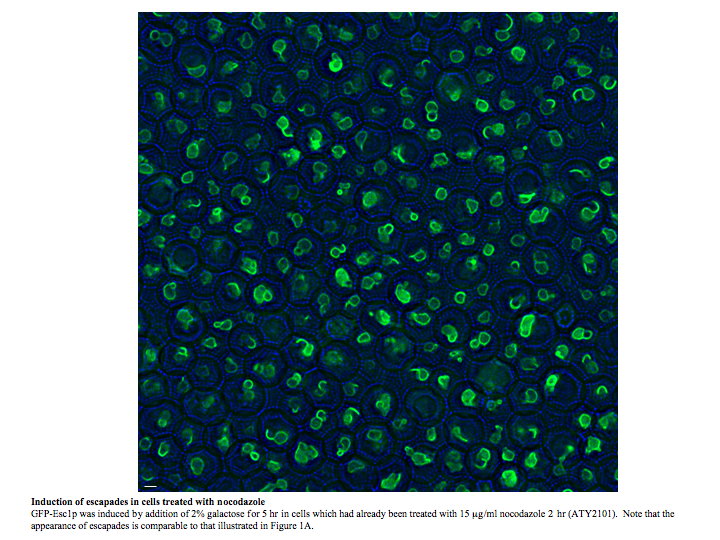

Supplement: Additional file 12 — Induction of escapades in cells treated with nocodazole. GFP-Esc1p was induced by addition of 2% galactose for 5 hr in cells which had already been treated with 15 μg/ml nocodazole 2 hr (ATY2101). Note that the appearance of escapades is comparable to that illustrated in Figure 1A. [file 1471-2121-8-47-S12.tiff]

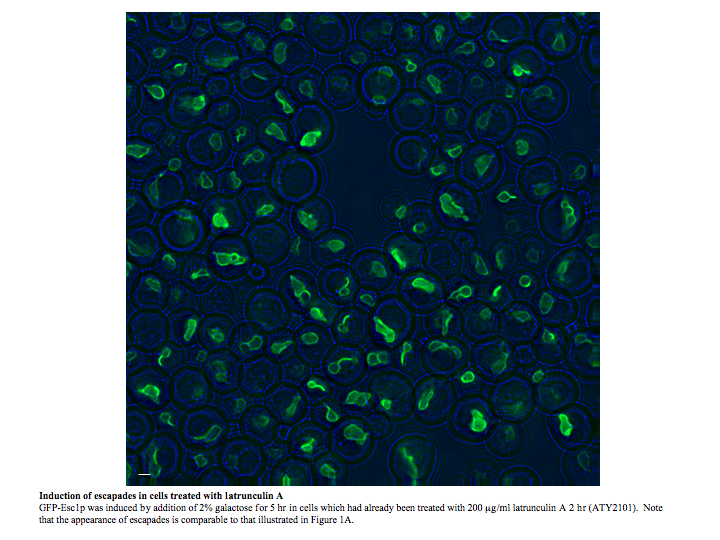

Supplement: Additional file 13 — Induction of escapades in cells treated with latrunculin A. GFP-Esc1p was induced by addition of 2% galactose for 5 hr in cells which had already been treated with 200 μg/ml latrunculin A 2 hr (ATY2101). Note that the appearance of escapades is comparable to that illustrated in Figure 1A. [file 1471-2121-8-47-S13.tiff]

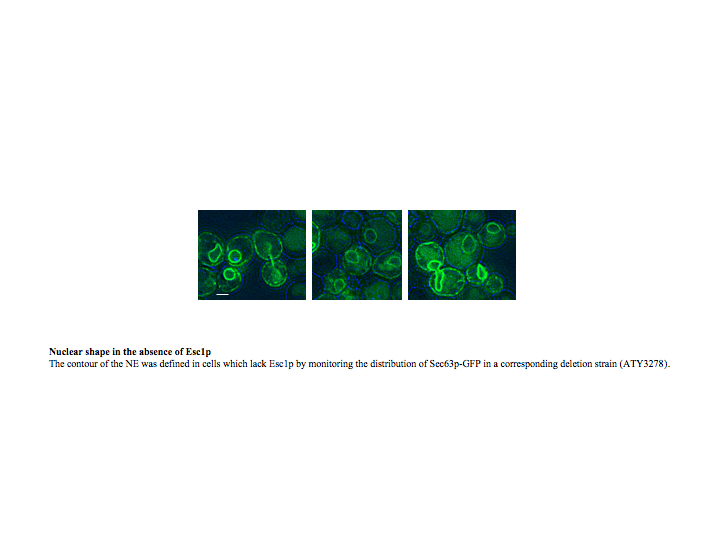

Supplement: Additional file 14 — Nuclear shape in the absence of Esc1p. The contour of the NE was defined in cells which lack Esc1p by monitoring the distribution of Sec63p-GFP in a corresponding deletion strain (ATY3278). [file 1471-2121-8-47-S14.tiff]
